# Tridecaptin-inspired antimicrobial peptides with activity against multidrug-resistant Gram-negative bacteria†
†Electronic supplementary information (ESI) available. See DOI: 10.1039/c9md00031c


**DOI:** 10.1039/c9md00031c

**Published:** 2019-03-12

**Authors:** Ross D. Ballantine, Conor E. McCallion, Elie Nassour, Sima Tokajian, Stephen A. Cochrane

**Affiliations:** a School of Chemistry and Chemical Engineering , David Keir Building , Queen's University Belfast , Stranmillis Road , Belfast , BT9 5AG , UK . Email: s.cochrane@qub.ac.uk; b Department of Natural Sciences , School of Arts and Sciences , Lebanese American University , Byblos , Lebanon

## Abstract

Antimicrobial peptides are a rich source of potential antibiotic candidates. The tridecaptins, a family of linear lipo-tridecapeptides, are easily synthesized and show strong activity against Gram-negative bacteria. However, their composition includes several expensive amino acids, such as d/l diaminobutyric acid and d-*allo*-isoleucine, significantly increasing their cost of synthesis. Herein, we report a series of new tridecaptin derivatives that are much cheaper to synthesize and retain strong activity against multidrug-resistant Gram-negative bacteria.

## Abstract 2

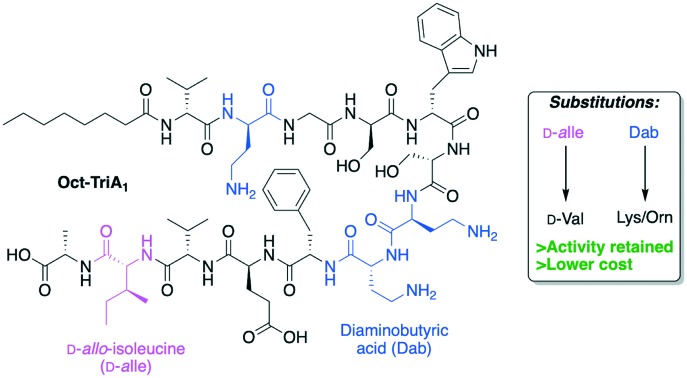
New tridecaptin analogues are cheaper to make and retain strong Gram-negative activity.

## Introduction

**Fig. 1:**
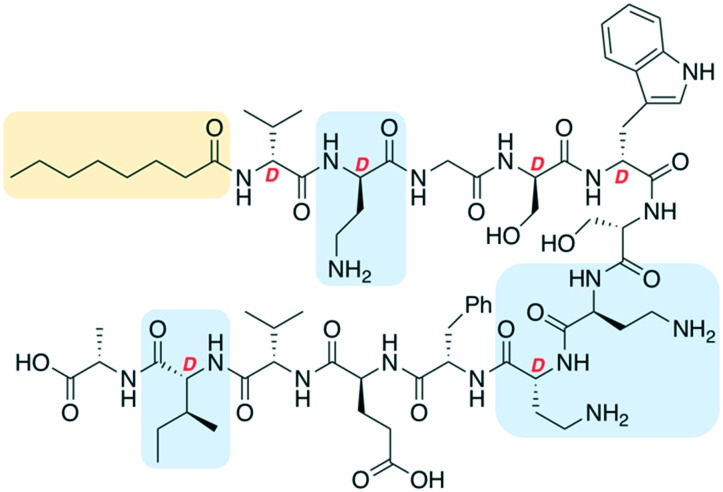
Structure of tridecaptin analogue Oct-TriA_1_ (**1**). d-Amino acids are labelled, the N-terminal lipid tail is highlighted in yellow and some of the most expensive non-proteinogenic amino acids are highlighted in blue.

One of the most significant challenges facing our generation is antimicrobial resistance (AMR).^1^–^3^ The human and financial cost of AMR is rising and failure to act could mean it causes more deaths than cancer by 2050.^4^ The World Health Organization has published a priority pathogens list, with the critical pathogens being carbapenem-resistant *Acinetobacter baumannii*, *Pseudomonas aeruginosa* and *Enterobacteriaceae*.^5^ These are all Gram-negative bacteria, which are significantly harder to treat than Gram-positive species as their outer-membrane blocks many antibiotics. Non-ribosomal peptides (NRPs) are attractive lead compounds in antibiotic development due to their large structural diversity. In recent years many NRPs have been discovered (or re-elucidated) that kill MDR bacteria, often through novel mechanisms of action.^6^,^7^ The tridecaptins are a family of linear NRPs that are N-terminally acylated and composed of 13 amino acids, more than half of which are non-proteinogenic residues (Fig. 1).^8^–^11^ They selectively kill Gram-negative bacteria by binding to the cell-wall precursor lipid II and disrupting the inner-membrane.^12^ This selective-binding was recently exploited to develop a tridecaptin-based fluorescent probe for differential staining of Gram-negative bacteria.^13^ It has also been shown that *Escherichia coli* does not develop resistance to octyl-tridecaptin A_1_ (Oct-TriA_1_) during continuous exposure over a 30 day period, highlighting its potential as an antibiotic lead candidate.^12^

The tridecaptins are produced by *Bacillus* and *Paenibacillus* species, however their production yields are low (<5 mg L^–1^).^8^,^9^ In contrast, solid-phase peptide synthesis (SPPS) can be used to prepare sizeable quantities of tridecaptins for structure–activity relationship (SAR) studies.^14^–^17^ A particular advantage that the tridecaptins have over many other antimicrobial NRPs is that they are not cyclic. This allows them to be easily assembled by SPPS without the need for orthogonal protecting group strategies and solution- or solid-phase macrocyclizations. However, a potential drawback in the chemical synthesis of the tridecaptins is the higher cost of some of the orthogonally-protected Fmoc-amino acids required, including Fmoc-Dab(Boc), Fmoc-d-Dab(Boc) and Fmoc-d-*allo*-Ile. If conservative substitutions could be made at positions containing these non-proteinogenic residues, the cost of synthesis would be significantly lowered. A previous alanine scan showed that several residues could be substituted with alanine in Oct-TriA_1_ without a significant loss of activity, however positions including d-Dab8 are vital.^18^ Herein we report our development of novel linear antimicrobial peptides based on the tridecaptin scaffold, that retain their activity against Gram-negative bacteria but are significantly cheaper to synthesize.

## Results and discussion

**Scheme 1:**
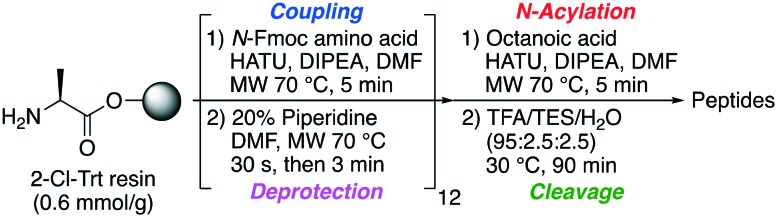
Automated SPPS workflow used to prepare tridecaptin analogues **1–8**. MW = microwave.

Oct-TriA_1_ (**1**) was first synthesized using automated Fmoc-SPPS (CEM Liberty 12 peptide synthesizer) (Scheme 1). Microwave assisted amino acid couplings and *N*-Fmoc deprotections allow these peptides to be efficiently assembled in reasonable overall yields (4–11%). Tridecaptin analogues are also readily synthesized by manual SPPS at room temperature, using 45 min couplings (HATU) and 3 × 1 min deprotections. Although more time consuming, we find the yield and crude purity of these peptides are superior to those synthesized by automated SPPS. After synthesis, the peptide is purified by reversed-phase (C_18_) high-performance liquid chromatography (RP-HPLC) for antimicrobial testing.

Previous studies have shown that the tridecaptins have strong activity against clinically relevant Gram-negative strains.^8^,^14^ This includes *A. baumannii* and *Enterobacteriaceae* such as *Klebsiella pneumoniae* and *Enterobacter* spp., however *P. aeruginosa* is less susceptible. The World Health Organization (WHO) currently lists carbapenemase-producing strains of these Gram-negative bacteria as critical priority pathogens. We therefore decided to synthesize a library of novel tridecaptin analogues containing conservative substitutions at positions 2, 7, 8 and/or 12, and evaluate their activity against a panel of these strains (Table 1). This panel of Gram-negative bacteria contains environmental samples isolated from sewage contaminated water in Lebanon, as well as several strains isolated from patients at clinical settings. The antibiotic susceptibility of these strains varies (see ESI†) but several of the clinical isolates are carbapenemase-producing organisms. In particular, *A. baumannii* ACM 11 and ACM 29 are extended-spectrum-β-lactamase-producing strains resistant to many β-lactam antibiotics, including the penicillins amoxicillin and ticarcillin, the carbapenems imipenem and ertapenem, and the cephalosporins cefalotin and cefuroxime. These strains are also resistant to several aminoglycosides and fluoroquinolones, although are susceptible to colicin.

**Table 1:** Antimicrobial activity of tridecaptin derivatives **1–8**

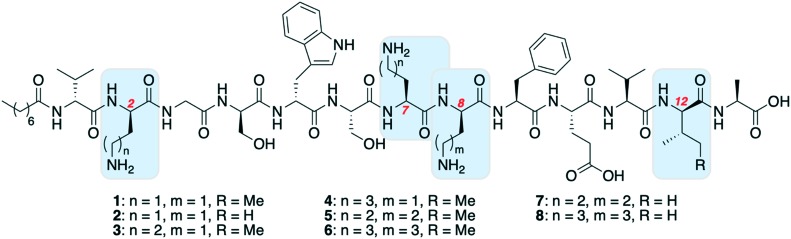										
Strain^*b*^	Isolate type	Carbapenemase-producing?	Peptide MIC^*a*^							
			**1**	**2**	**3**	**4**	**5**	**6**	**7**	**8**
*A. baumannii*	Environmental	No	12.5	25	100	50	12.5	6.25	25	>100
*A. baumannii* ACM 11	Clinical	Yes	25	100	50	25	50	25	25	>100
*A. baumannii* ACM 29	Clinical	Yes	25	50	50	50	50	25	50	>100
*E. cloacae*	Clinical	Yes	3.13	6.25	25	25	6.25	25	12.5	>100
*K. pneumoniae*	Environmental	No	6.25	12.5	100	50	12.5	50	50	>100
*K. pneumoniae* IMP 170	Clinical	Yes	6.25	12.5	25	25	25	50	25	>100
*K. pneumoniae* IMP 177	Clinical	Yes	6.25	25	25	25	25	25	12.5	>100
*K. pneumoniae* IMP 204	Clinical	Yes	12.5	12.5	25	25	25	50	12.5	>100
*K. pneumoniae* IMP 216	Clinical	Yes	6.25	12.5	25	25	6.25	25	12.5	>100
*K. pneumoniae* IMP 485	Clinical	Yes	6.25	12.5	25	25	25	25	12.5	>100
*P. pseudoalcaligenes*	Environmental	No	50	50	12.5	25	50	100	6.25	>100

^*a*^MIC = minimum inhibitory concentration. Determined by microbroth dilutions assays and experiments run in duplicate. Values are shown to three significant figures and reported in μg mL^–1^.

^*b*^Additional strain information including antibiotic susceptibility and isolation data are available in the ESI.

The first amino acid substitution we explored was replacement of d-*allo*-Ile12 with d-Val, which occurs in natural TriA_2_ variants. Fmoc-d-Val is ∼30 times less expensive than Fmoc-d-*allo*-Ile (according to Fluorochem pricing). The antimicrobial activity of Oct-TriA_2_ (**2**) is two- to four-fold less active than Oct-TriA_1_ (**1**) against most strains tested. The minimum inhibitory concentrations (MICs) of Oct-TriA_1_ against *K. pneumoniae* and *E. cloacae* strains were typically 6.25 μg mL^–1^ but lower against carbapenemase-producing *A. baumannii* strains (25 μg mL^–1^). These results suggest that an additional methyl unit at position 12 has a small but positive contribution to the antimicrobial activity.

We next proceeded to replace the d-Dab and l-Dab residues, found at positions 2 and 8, and 7 respectively. Longer chain basic amino acids, such as Lys and Orn, are significantly cheaper than their Dab counterparts. It was previously shown that d-Dab8 is essential for antimicrobial activity,^18^ and an experimentally derived model of Oct-TriA_1_ bound to its cellular receptor, the peptidoglycan intermediate lipid II, supports this observation.^12^ Therefore we first prepared peptides in which only the amino acids at positions 2 and 7 were substituted with Lys or Orn (d/l configuration retained). Oct-TriA_1_ (2-d-Orn, 7-Orn) (**3**) and Oct-TriA_1_ (2-d-Lys, 7-Lys) (**4**) have comparable antimicrobial activity but are two- to four-fold less active than Oct-TriA_1_ against all of the *Acinetobacter* and *Enterobacteriaceae* spp. tested. However, both peptides **3** and **4** have enhanced antimicrobial activity against *P. pseudoalcaligenes*. Encouraged by these results, we then proceeded to synthesize and test variants in which d-Dab8 had also been substituted. Oct-TriA_1_ (2,8-d-Orn, 7-Orn) (**5**) and Oct-TriA_1_ (2,8-d-Lys, 7-Lys) (**6**) also retain their Gram-negative activity, with comparable MICs to peptides **3** and **4** against most strains. Finally, the Lys/Orn substitutions at the 2, 7 and 8 positions were also incorporated into TriA_2_ analogues, yielding Oct-TriA_2_ (2,8-d-Orn, 7-Orn) (**7**) and Oct-TriA_2_ (2,8-d-Lys, 7-Lys) (**8**). At this stage the triple Oct-TriA_2_ mutant **8** showed no antimicrobial activity at the highest concentrations tested (100 μg mL^–1^). Gratifyingly, Oct-TriA_2_ (2,8-d-Orn, 7-Orn) (**7**) showed comparable activity to Oct-TriA_1_ against the *A. baumannii* strains and four-fold enhanced activity against *P. pseudoalcaligenes*, although was two- to four-fold less active against the *K. pneumoniae* strains.

Some general conclusions can be drawn from these results. Firstly, Oct-TriA_1_ (**1**) has the strongest antimicrobial activity in the majority of cases, except against *P. pseudoalcaligenes*. Secondly, Oct-TriA_2_ analogues are two- to four-fold less active than their Oct-TriA_1_ counterparts in most cases. Thirdly, substitution of d-Dab8 with d-Lys is more detrimental to activity than substitution with d-Orn. Mechanistically, this may be related to lipid II binding by the tridecaptins, however further *in vitro* studies using lipid II are required to confirm this and are beyond the scope of the present study. Fourthly, Oct-TriA_2_ (2,8-d-Orn, 7-Orn) (**7**) is the most promising variant and although less active against some strains, it is significantly cheaper to synthesize than Oct-TriA_1_.

Having assessed the antimicrobial activity of peptides **1–8** we next proceeded to assess their haemolytic activities (Table 2). Haemolytic activity was tested at 100 μg mL^–1^, as the concentration required for haemolysis should be higher than antimicrobial MICs. Equine erythrocytes were exposed to peptides **1–8** and incubated for 30 min at 37 °C (Table 2). The Oct-TriA_2_ analogues are significantly less haemolytic than their Oct-TriA_1_ counterparts. >50% haemolysis was observed for most peptides, with the exception of Oct-TriA_2_ (2,8-d-Orn, 7-Orn) (**7**) and Oct-TriA_2_ (2,8-d-Lys, 7-Lys) (**8**). The low haemolytic activity observed for Oct-TriA_2_ (2,8-d-Lys, 7-Lys) (**8**) is consistent with its poor antimicrobial activity. In contrast, Oct-TriA_2_ (2,8-d-Orn, 7-Orn) (**7**), which is one of the most potent new tridecaptin analogues, has lower haemolytic activity (39.8%) than the remaining peptides. This is promising, as it is also significantly cheaper to synthesize than Oct-TriA_1_.

**Table 2:** Haemolytic activity of tridecaptin derivatives **1–8**

Peptide^*a*^	% haemolysis^*b*^
**1**	89.6
**2**	52.1
**3**	66.7
**4**	71.4
**5**	77.5
**6**	86.4
**7**	39.8
**8**	2.8

^*a*^All peptides were tested at 100 μg mL^–1^.

^*b*^Experiments were run in triplicate. Absorbance was measured at 415 nm and percent haemolysis of the peptides were calculated relative to Triton X-100 (taken as 100%).

In summary, we have synthesized novel antimicrobial peptides based on the tridecaptins, wherein the most expensive residues are replaced with cheaper analogues. Most of these synthetic peptides retain activity against Gram-negative bacteria. In particular, Oct-TriA_2_ (2,8-d-Orn, 7-Orn) (**7**), displayed strong activity against MDR strains (clinical and environmental isolates) of *A. baumannii*, *K. pneumoniae* and *E. cloacae*, several of which are carbapenem-resistant. Peptide **7** was also four-fold more active against *P. pseudoalcaligenes* than the canonical Oct-TriA_1_, has lower haemolytic activity and is much cheaper to synthesize. Given the need for new antimicrobials against carbapenem resistant Gram-negative bacteria, Oct-TriA_2_ (2,8-d-Orn, 7-Orn) may prove a good compound for further development.

## Conflicts of interest

There are no conflicts to declare.

## Supplementary Material

Supplementary information
