# Supplementary material for: Tridecaptin-inspired antimicrobial peptides with activity against multidrug-resistant Gram-negative bacteria
Source: Medchemcomm. 2019 Mar 12;10(3):484–7. doi: 10.1039/c9md00031c (PMC6457190; doi:10.1039/c9md00031c)
Supplement: Supplementary file 1 [file MD-010-C9MD00031C-s001.pdf]

Electronic Supplementary Material (ESI) for MedChemComm.
This journal is © The Royal Society of Chemistry 2019

Supplementary Information

FOR

Tridecaptin-Inspired Antimicrobial Peptides with Activity Against Multidrug-Resistant Gram-Negative Bacteria

Ross D. Ballantine,[a] Conor E. McCallion,[a] Elie Nassour,[b] Sima Tokajian[b] and Stephen A. Cochrane*[a]

[a] School of Chemistry and Chemical Engineering, David Keir Building, Stranmillis Road, Queen's University Belfast, Belfast, UK, BT9 5AG; [b] Department of Natural Sciences, School of Arts and Sciences, Lebanese American University, Byblos, Lebanon

*To whom correspondence should be addressed. Email: s.cochrane@qub.ac.uk

I. Materials

All Proteinogenic Fmoc-amino acids used in this study were purchased from CEM. The remaining Fmoc-amino acids, including Fmoc-Dab(Boc)-OH, Fmoc-D-Dab(Boc)-OH, Fmoc-D-*a*Ile-OH, Fmoc-D-Lys(Boc)-OH, Fmoc-Orn(Boc)-OH, Fmoc-D-Orn(Boc)-OH, Fmoc-D-Trp(Boc)-OH and Fmoc-D-Val-OH were purchased from Fluorochem. [Bis(dimethylamino)methylene]-1*H*-1,2,3-triazolo[4,5-*b*]pyridinium-3-oxidhexafluorophosphate (HATU), trifluoroacetic acid (TFA) and triisopropylsilane (TIPS) were also purchased from Fluorochem. Ala-2-chlorotrityl resin and diisopropylethylamine (DIPEA) were purchased from Sigma-Aldrich. HPLC grade acetonitrile (ACN), dichloromethane (DCM) and dimethylformamide (DMF) were purchased from Merck. All chemicals were used without further purification.

II. General procedure for automated peptide synthesis

Peptides were synthesized on a CEM Liberty 12 Microwave Peptide Synthesizer. Solid phase synthesis was carried out on a 0.1 mmol scale using Fmoc chemistry on 2-chlorotrityl (2CT) resin preloaded with Ala (0.6 mmol/g). Factory settings were used for all coupling and deprotection cycles. Asymmetrically protected amino acids were used as 0.2 M solutions in DMF, with amino acid subunits being coupled using HATU as the activator and DIPEA as the activator base and heated to 70 °C for 5 min. Fmoc residues were deprotected using a 20 % solution of piperidine in DMF. An initial deprotection was performed at 70 °C for 0.5 min, followed by an additional deprotection at 70 °C for 3 min. Upon completion of synthesis, the peptide resin was washed with DCM (3 x 5 mL) in a glass-fritted filter funnel and dried under vacuum suction for 15 min. Dried peptide resin was transferred to a vial containing TFA:TIPS:H_2O (95:2.5:2.5, 10 mL) and heated at 30 °C for 1.5 h with periodic manual shaking. The resin beads were then removed by filtration through glass wool and the filtrate was concentrated *in vacuo*. Et_2O (5 mL) was added to precipitate crude peptide and the resulting suspension transferred to 2 mL centrifuge tubes. The tubes were centrifuged for 1 min using a microcentrifuge and the liquid decanted. Pellets were resuspended in Et_2O (1 mL), vortexed, centrifuged and the liquid decanted to yield crude peptide pellets.

III. Purification and analysis of peptides

Crude peptide was dissolved in minimal 1:1 0.1% aqueous TFA/ACN and purified by Reversed-Phase High Performance Column Chromatography (RP-HPLC). Purification was performed on a Perkin Elmer HPLC system composed of a 200 series binary pump, UV/Vis detector, vacuum degasser, Rheodyne 7725i injector equipped with a 2 mL sample loop and Phenomenex Luna C18 column (5 µm, 250 x 21.2 mm). The system was operated using ThermoFisher Chromeleon 7.2 software. Runs were performed at a flow rate of 10 mL/min with UV detection at 220 nm. Solvent A = 0.1 % TFA in MilliQ water and solvent B = 0.1 % TFA in ACN. A gradient method was employed, starting from 20 % B and 80 % A for 5 min, ramping up to 55 % B over 30 min, then ramping up to 95 % B over 3 min, remaining at 95 % B for 3 min, ramping down to 20 % B over 2 min before staying at 20 % B for 5 min. Product containing fractions were pooled, partially concentrated under vacuum, frozen and then lyophilized to yield pure peptides as white flocculent solids. A small amount of purified peptide was analyzed by analytical HPLC. Analytical runs were performed on a Perkin Elmer HPLC system composed of a 200 series quaternary pump, UV/Vis detector, vacuum degasser, Rheodyne 7725i injector equipped with a 200 µL sample loop and Phenomenex Luna C18 column (5 µm, 150 x 4.6 mm). The system was operated using ThermoFisher Chromeleon 7.2 software. Runs were performed at a flow rate of 2 mL/min with UV detection at 220 nm. Solvent A = 0.1 % TFA in

MilliQ water and solvent B = 0.1 % TFA in ACN. A gradient method was employed, starting from 20 % B and 80 % A for 2 min, ramping up to 95 % B over 18 min, ramping down to 20 % B over 0.1 min before staying at 20 % B for 3.9 min. High resolution mass spectrometry (HRMS) spectra of all peptides were recorded by Analytical Services and Environmental Projects (ASEP) at Queen's University Belfast on a Waters LCT Premier ToF mass spectrometer using the electrospray ionisation (ESI) technique.

IV. Antimicrobial testing

All minimum inhibitory concentrations (MICs) were determined according to Clinical and Standards Laboratory Institute (CLSI) guidelines. Briefly, peptides were dissolved in Muller-Hinton Broth (MHB) and serial dilutions made across a 96 well plate. Each well was inoculated with a suspension of the required bacterial strain to reach a final inoculum of 5 x 10^5 colony forming units per mL. The MIC was taken as the lowest concentration with no visible growth after 18 hours.

V. Additional information on multidrug resistant strains

Antimicrobial susceptibility testing was performed using the disk diffusion method and the results were interpreted according to the Clinical & Laboratory Standards Institute (CLSI) guidelines.

Organism	Resistant	Susceptible
Acinetobacter baumannii<br><br>Isolated from sewage contaminated water (Tripoli, Lebanon).	Amoxicillin	Tazobactam
	Aztreonam	Ticarcillin
	Cefotaxime	Imipenem
	Cefuroxime	Meropenem
	Tetracycline	Colistin
	Ofloxacin	Tobramycin
		Gentamycin

Acinetobacter baumannii ACM 11<br><br>Isolated from DTA of 64-year-old female.	Gentamicin	Colistin
	Amikacin	
	Tazobactam	
	Ceftazimide	
	Cefepime	
	Imipenem	
	Meropenem	
	Ciprofloxacin	
	Levofloxacin	

Acinetobacter baumannii ACM 29<br><br>Isolated from peritoneum of 3-year-old male.	Gentamicin	Colistin
	Amikacin	
	Tazobactam	
	Ceftazimide	
	Cefepime	
	Imipenem	
	Meropenem	
	Ciprofloxacin	
	Levofloxacin	

Enterobacter cloacae<br><br>Isolated from stool of a female patient (age not specified).	Amoxicillin	Amikacin
	Ticarcillin	Fosfomycin E
	Tazobactan	Colistin
	Imipenem	
	Ertapenem	
	Cefalotin	
	Cefuroxime	
	Ceftazimide	
	Tobramycin	
	Gentamycin	
	Ofloxacin	
	Cirpofloxacin	
	Levofloxacin	

Klebsiella pneumoniae<br><br>Isolated from sewage contaminated water (Tripoli, Lebanon).	Amoxicillin	Imipenem
	Ampicillin	Ertapenem
	Ticarcillin	Cefoxitin
	Tazobactam	Nitrofurantoin
	Aztreonam	Colistin
	Cefotaxime	Amikacin
	Cefuroxime	Meropenem
	Ceftriaxone	
	Ceftazidime	
	Cefepime	
	Tobramycin	
	Gentamycin	
	Ofloxacin	

Klebsiella pneumoniae IMP 170<br><br>Isolated from DTA of 67-year-old male.	Ertapenem	Amikacin
	Imipenem	Fosfomycin
	Ampicilin	Tigecycline
	Augmentin	Gentamicin
	Aztreonam	
	Cefamandole	

Klebsiella pneumoniae IMP 177<br><br>Isolated from cyst of 20-year-old male.	Ampicilin	Amikacin
	Augmentin	Fosfomycin
	Tazobactam	Tigecycline
	Aztreonam	Gentamicin
	Cefamandole	Norfloxacin
	Ceftazimide	Tetracycline
	Cefixime	

Klebsiella pneumoniae IMP 204<br><br>Isolated from urine of 73-year-old male.	Ertapenem	Amikacin
	Imipenem	Gentamicin
	Meropenem	Aztreonam
	Fosfomycin	Cefepime
	Ampicilin	Ceftazimide
	Augmentin	

Klebsiella pneumoniae IMP 216<br><br>Isolated from urine of 57-year-old female.	Ertapenem	Meropenem
	Imipenem	Fosfomycin
	Amikacin	
	Gentamicin	
	Ampicillin	
	Augmentin	
	Tazobactam	
	Aztreonam	

Klebsiella pneumoniae IMP 485<br><br>Isolated from skin of 59-year-old male.	Ertapenem	Amikacin
	Imipenem	Fosfomycin
	Meropenem	Tigecycline
	Ampicilin	Gentamicin
	Augmentin	Aztreonam
	Tazobactam	Cefamandole
	Trimehtoprim	Cefoxitin

Pseudomonas pseudoalcaligenes<br><br>Isolated from sewage contaminated water (Saida, Lebanon).	Amoxicillin	Imipenem
	Ampicillin	Ertapenem
	Ticarcillin	Tobramycin
	Cefotaxime	Gentamycin
	Cefuroxime	Tetracycline
		Ceftriaxone

VI. Haemolytic activity assays

A 96-well plate was first prepared to contain phosphate-buffered saline (PBS) solutions (100 µL) of tridecaptin analogues **1** – **8** (200 µg/mL) (experiments run in triplicate). 0.1 % Triton X-100 (100 µL) was used as a positive control, while PBS (100 µL) was used as a blank. After the plate had been prepared, defibrinated horse blood (0.5 mL, VWR International) was diluted with phosphate-buffered saline (9.5 mL) and gently shaken. The resulting mixture was centrifuged (5600 rpm, 5 min) using a Thermo Scientific Heraeus Labofuge 200 centrifuge and the supernatant carefully removed. This process was repeated three more times to remove any free hemoglobin. The resulting erythrocyte pellet was gently resuspended in 9.5 mL of PBS. Aliquots of the resulting mixture (100 µL) were added to the appropriate wells on the 96-well plate using a multichannel pipette and the plate incubated at 37 °C for 30 min. The samples were gently mixed by pipetting, and 20 µL of each was added to 200 µL of PBS. Once again, the samples were gently mixed. The diluted samples were then centrifuged (7000 rpm, 5 min) using a SciSpin MINI Microfuge. Supernatant (150 µL) from each sample was added to a new 96-well plate. The absorbance of each sample was individually measured at 415 nm using a Denovix DS-11 FX+ Spectrophotometer. Percent hemolysis of the peptides was calculated relative to Triton X- 100, while the PBS negative control was used as a blank.

VII. HPLC and HRMS analysis of peptides

Peptide	Name	Chemical Formula	Calcd Exact Mass	Mass found	Calcd	Overall Yield [%]
**1**	Oct-TriA$_1$	$C_{72}H_{113}N_{17}O_{19}$	1519.8399	760.9263 [M + 2H]$^{2+}$	760.9278	11
**2**	Oct-TriA$_2$	$C_{71}H_{111}N_{17}O_{19}$	1505.8242	1504.8159 [M - H]$^{-}$	1504.8169	5
**3**	Oct-TriA$_1$(2-D-Orn, 7-Orn)	$C_{74}H_{117}N_{17}O_{19}$	1547.8712	1548.8785 [M + H]$^{+}$	1548.8736	6
**4**	Oct-TriA$_1$(2-D-Lys, 7-Lys)	$C_{76}H_{121}N_{17}O_{19}$	1575.9025	1576.9103 [M + H]$^{+}$	1576.9098	5
**5**	Oct-TriA$_1$(2,8-D-Orn, 7-Orn)	$C_{75}H_{119}N_{17}O_{19}$	1561.8868	1562.8923 [M + H]$^{+}$	1562.8868	6
**6**	Oct-TriA$_1$(2,8-D-Lys, 7-Lys)	$C_{78}H_{125}N_{17}O_{19}$	1603.9338	1602.9294 [M - H]$^{-}$	1602.9265	6
**7**	Oct-TriA$_2$(2,8-D-Orn, 7-Orn)	$C_{74}H_{117}N_{17}O_{19}$	1547.8712	795.9669 [M + 2H]$^{2+}$	795.9677	4
**8**	Oct-TriA$_2$(2,8-D-Lys, 7-Lys)	$C_{77}H_{123}N_{17}O_{19}$	1589.9181	774.9443 [M + 2H]$^{2+}$	774.9434	4

Table S1. Peptide number, name, chemical formula, exact mass, mass found and overall yield for peptides **1** – **8**.

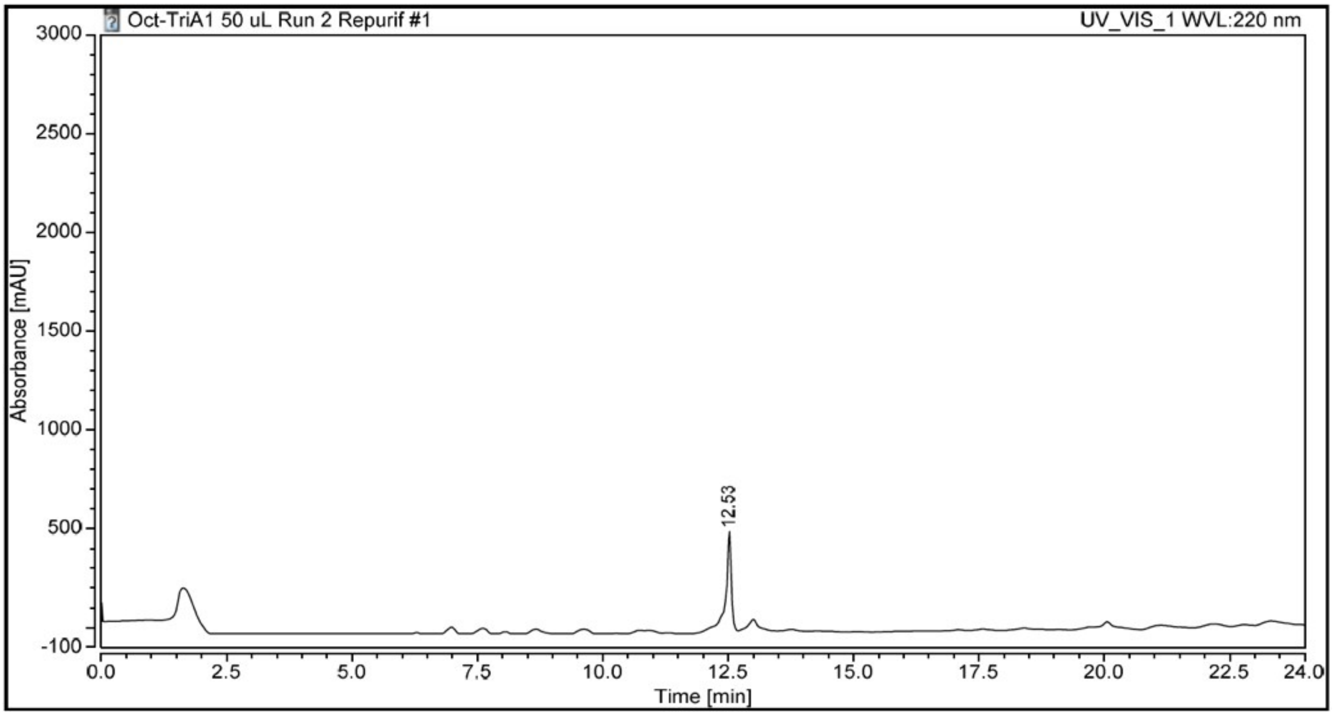

Fig. S1. HPLC trace showing the reinjection of purified Oct-TriA$_1$ (**1**). The peptide eluted as a single peak at 12.53 min using the HPLC method outlined in part III.

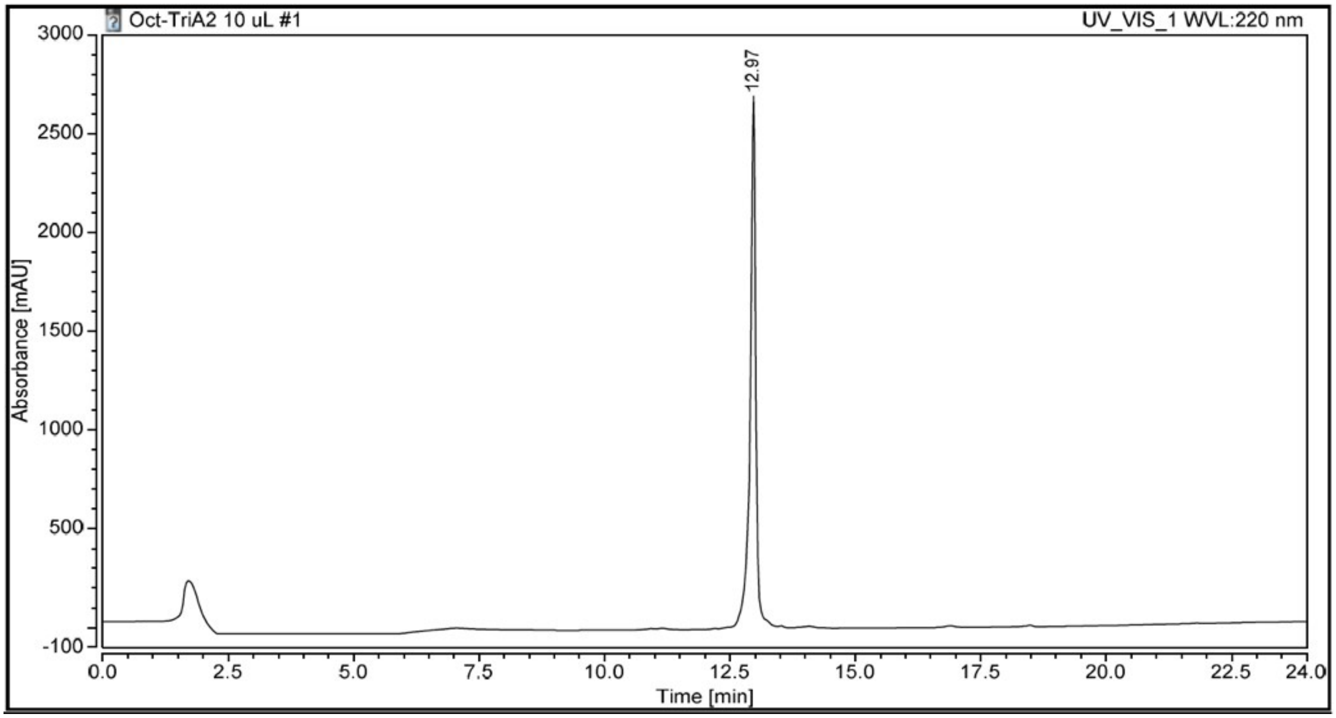

Fig. S2. HPLC trace showing the reinjection of purified Oct-TriA$_2$ (**2**). The peptide eluted as a single peak at 12.97 min using the HPLC method outlined in part III.

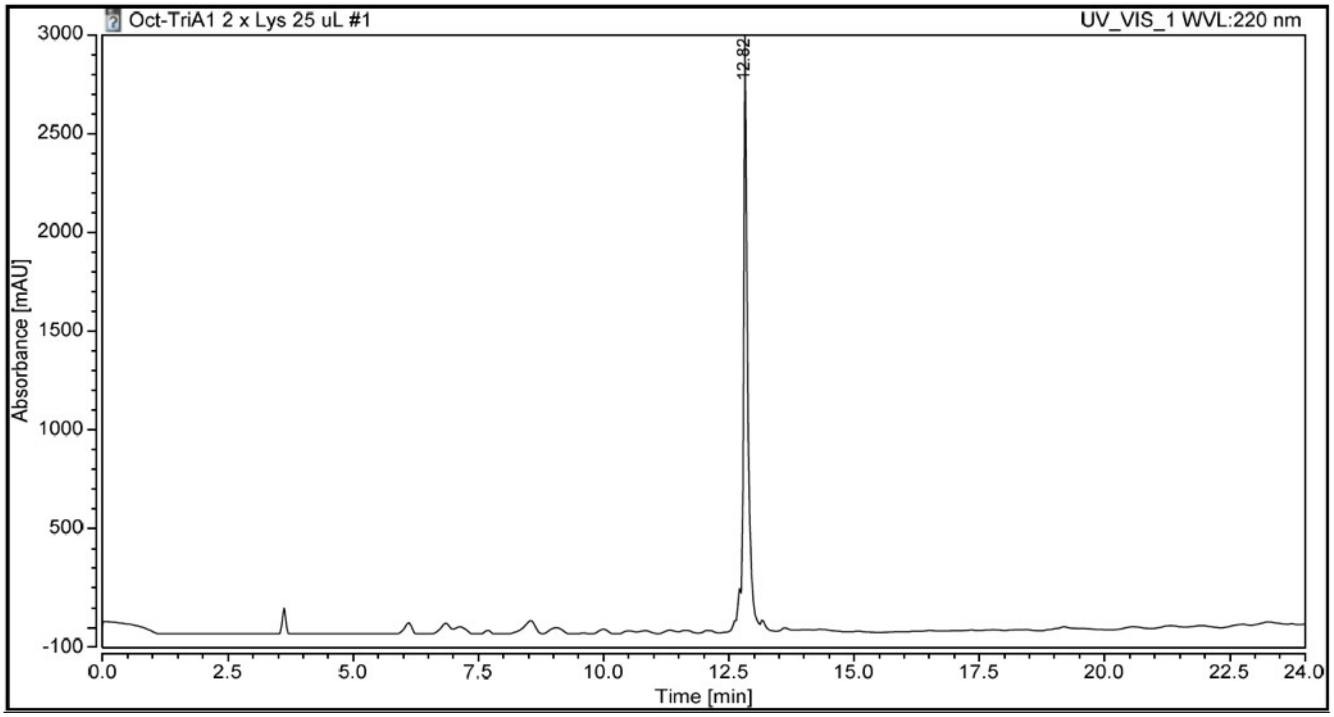

Fig. S3. HPLC trace showing the reinjection of purified Oct-TriA$_1$(2-D-Orn, 7-Orn) (**3**). The peptide eluted as a single peak at 12.82 min using the HPLC method outlined in part III.

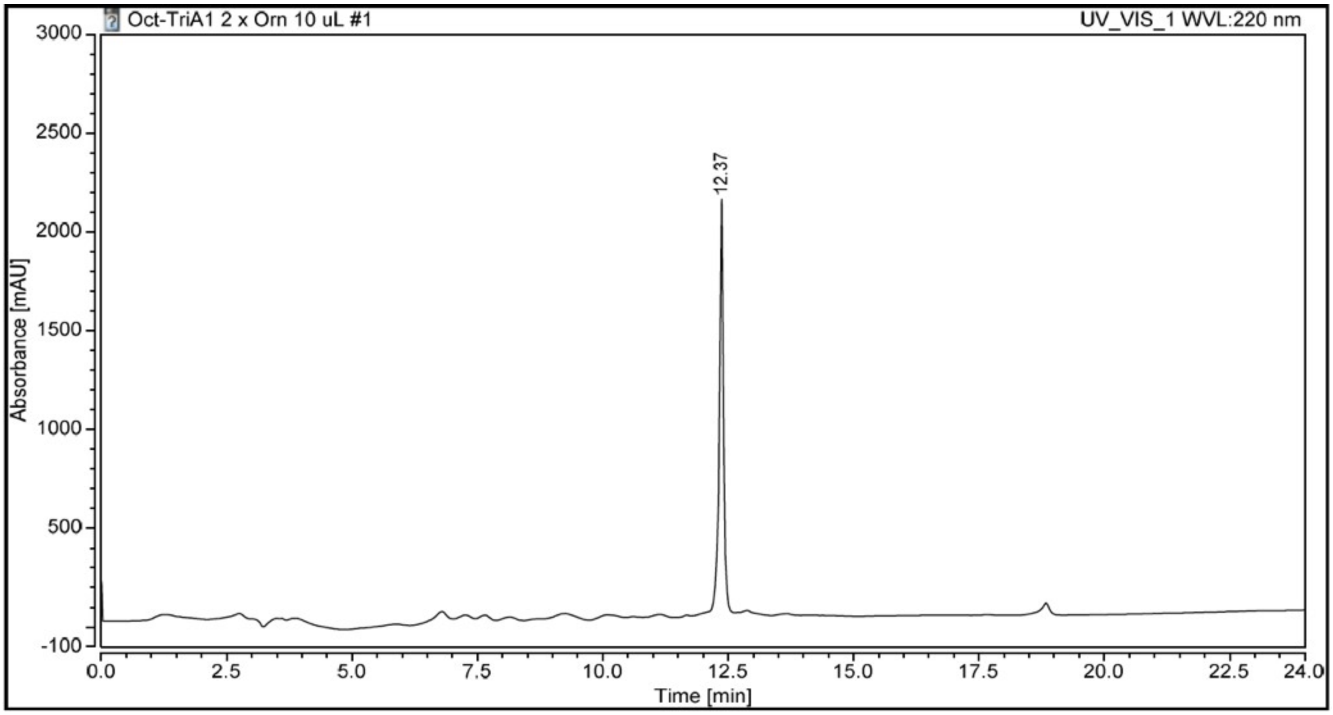

Fig. S4. HPLC trace showing the reinjection of purified Oct-TriA$_1$(2-D-Lys, 7-Lys) (**4**). The peptide eluted as a single peak at 12.37 min using the HPLC method outlined in part III.

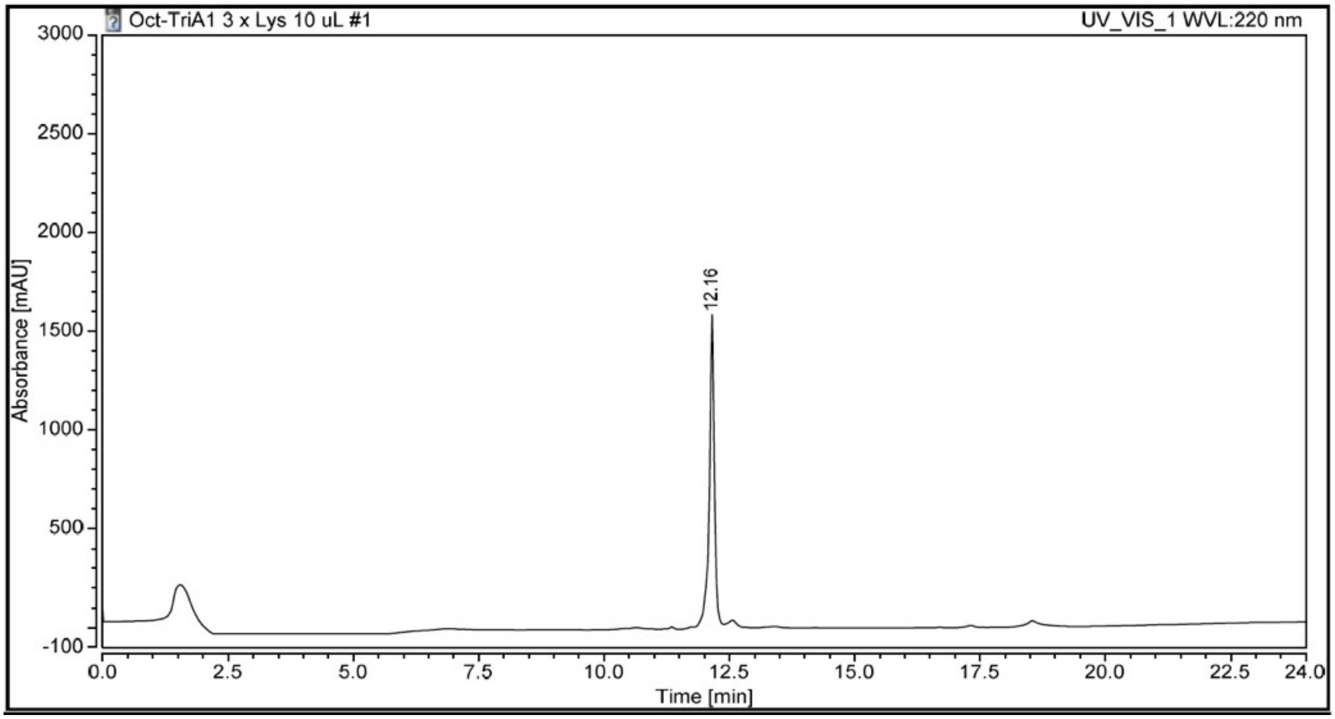

Fig. S5. HPLC trace showing the reinjection of purified Oct-TriA$_1$(2,8-D-Orn, 7-Orn) (**5**). The peptide eluted as a single peak at 12.16 min using the HPLC method outlined in part III.

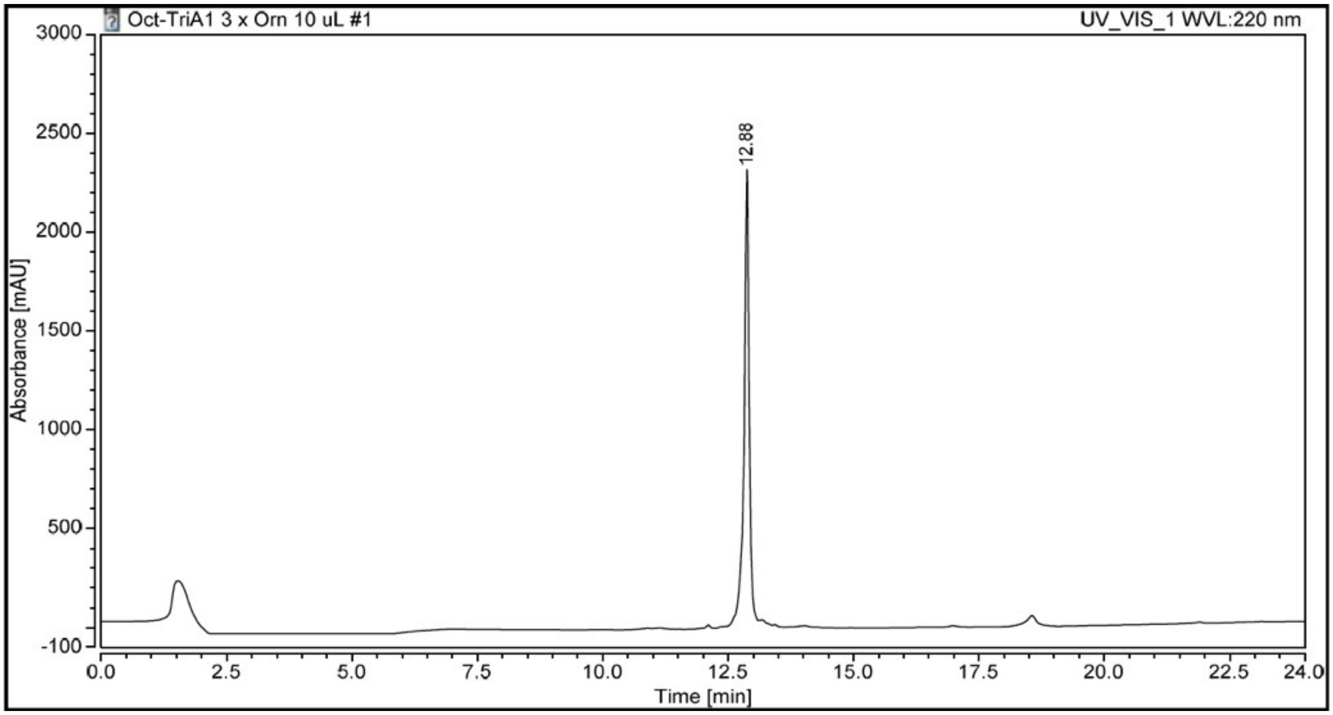

Fig. S6. HPLC trace showing the reinjection of purified of Oct-TriA$_1$(2,8-D-Lys, 7-Lys) (**6**). The peptide eluted as a single peak at 12.88 min using the HPLC method outlined in part III.

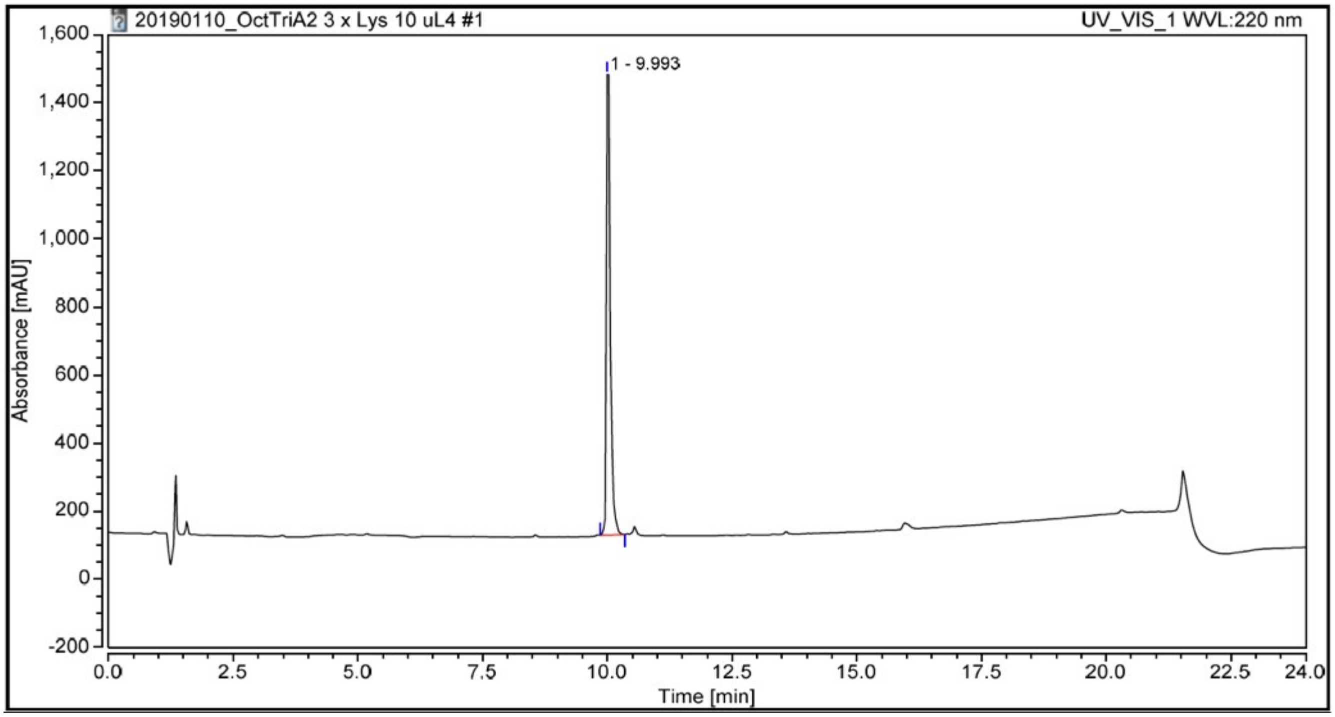

Fig. S7. HPLC trace showing the reinjection of purified Oct-TriA$_2$(2,8-D-Orn, 7-Orn) (**7**). The peptide eluted as a single peak at 9.99 min using the HPLC method outlined in part III.

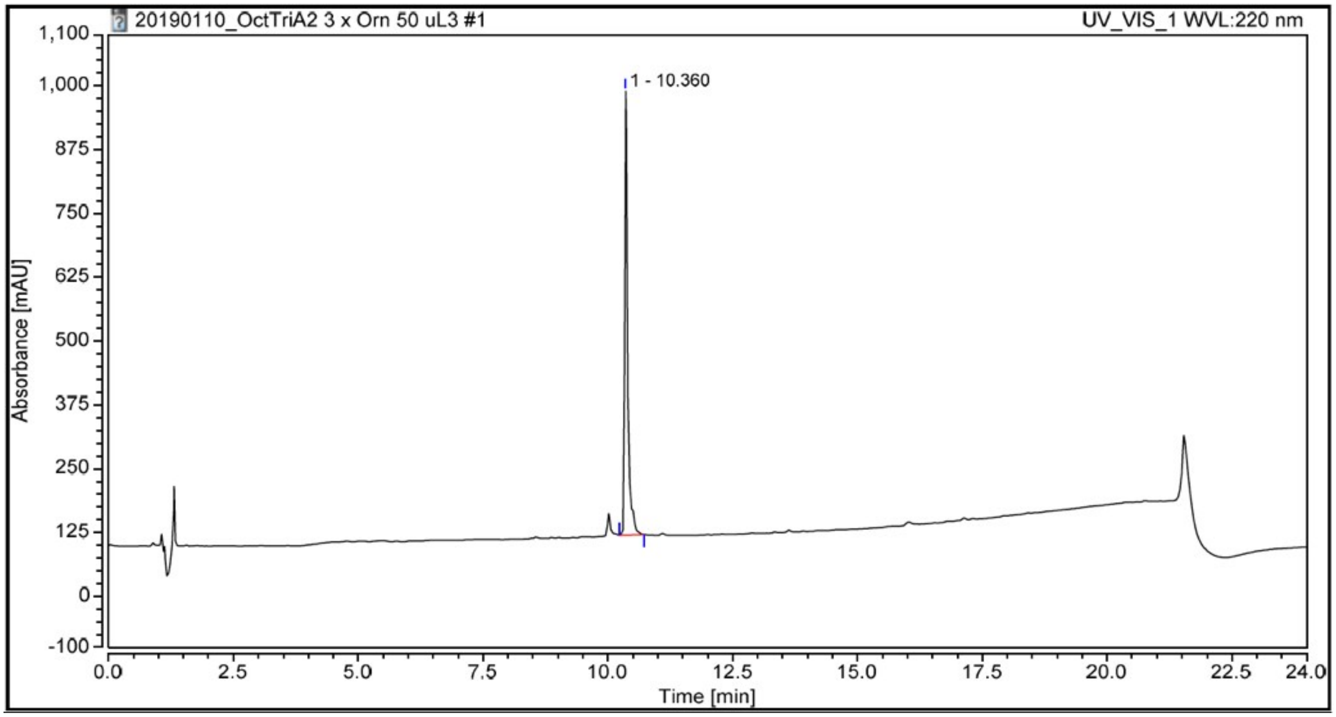

Fig. S8. HPLC trace showing the reinjection of purified Oct-TriA$_2$(2,8-D-Lys, 7-Lys) (**8**). The peptide eluted as a single peak at 10.36 min using the HPLC method outlined in part III.
